# Dual Portal Closure of Periportal Capsulotomy for Hip Arthroscopy

**DOI:** 10.1002/atn2.70098

**Published:** 2026-04-30

**Authors:** Benjamin Lurie, Joseph M. Sliepka, Nyaluma N. Wagala, Alan L. Zhang

**Affiliations:** ^1^ Department of Orthopaedic Surgery University of California‐San Francisco San Francisco California U.S.A.

## Abstract

Capsular management in hip arthroscopy is an area of ongoing research, where ease of access to the hip joint is balanced with the preservation of capsular integrity and stability. Traditional techniques such as interportal and T capsulotomies allow broad exposure but may increase the risk of postoperative instability or microinstability if not repaired. Periportal capsulotomy aims to limit disruption of the iliofemoral ligament while still allowing sufficient access for safe and efficient treatment of hip pathology and typically does not necessitate closure. However, in patients who are at higher risk for postoperative instability such as those with borderline dysplasia or joint hypermobility, closure of the periportal capsulotomy may be warranted. We describe a technique for intracapsular closure of both the anterolateral and modified midanterior periportal capsulotomies in which closure of the anterolateral portal capsulotomy is facilitated by passage of the capsule closure suture at the beginning of the case while the hip is in traction. This approach minimizes the risk of postoperative instability without significant additional operative time.

## Graphical abstract

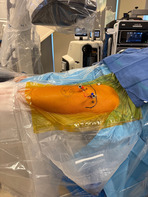

**VIDEO 1:** This video describes our technique for dual portal closure after periportal capsulotomy in hip arthroscopy. The patient is positioned supine with postless table attachment to allow for distraction of the left hip joint while minimizing the risk of post‐related complications. Two portals are used: the anterolateral and the modified midanterior portal. Using radiofrequency ablation, the modified midanterior portal (which resides within the iliofemoral ligament) is dilated to 8 to 10 mm. The anterolateral portal is dilated to 6 to 8 mm. The portals are primarily dilated in line with one another in case they would need to be extended into an interportal capsulotomy. Next, the suture for repair of the anterolateral capsulotomy is placed. The 70° arthroscope is then moved to the modified midanterior portal, and the anterolateral cannula is exchanged for a sled. A #2 Orthocord suture is placed across the anterolateral periportal capsulotomy using a Stryker 70° Slingshot suture passer. The suture is snapped and left untied until the end of the case. After completing the labral repair, acetabuloplasty, and femoroplasty, the camera is placed in the anterolateral portal, the disposable cannula in the modified midanterior portal is withdrawn until it is superficial to the capsule, and the 70° suture passer is used to pass a stitch across the capsulotomy. The stitch is then tied and cut with a closed cutter. The cannulas and arthroscope are then completely removed from the patient and the anterolateral sutures are tied blind and cut with a closed cutter. Video content can be viewed at https://doi.org/10.1002/atn2.70098.

Capsular management during hip arthroscopy is an active area of research and debate. There are numerous capsulotomy techniques for accessing the hip joint, including interportal capsulotomy, T capsulotomy, periportal capsulotomy, and puncture capsulotomy. Interportal capsulotomy is one of the more common capsular management techniques, during which the capsule and iliofemoral ligament is incised between the anterolateral (AL) and midanterior (MA) portals.^1^, ^2^ The T capsulotomy affords additional exposure and is often employed for the resection of large and distal cam lesions. This technique adds a longitudinal limb along the long axis of the femoral neck.^2^, ^3^, ^4^ Periportal capsulotomy helps preserve the iliofemoral ligament by dilating the AL and modified MA portals without connecting them as in an interportal capsulotomy.^5^, ^6^ Finally, puncture capsulotomy similarly attempts to limit injury to capsular tissue and involves the use of multiple accessory portals to provide access to different parts of the hip joint.^7^

When an interportal or T capsulotomy is performed, recent literature has generally supported capsule closure to reduce the risk of instability or microinstability after hip arthroscopy.^8^, ^9^, ^10^, ^11^ The periportal capsulotomy technique was devised to preserve the iliofemoral ligament while allowing sufficient access to perform a safe and efficient labral repair, acetabuloplasty, and femoroplasty.^5^, ^12^, ^13^ This technique dilates 2 openings in the capsule, 1 from the modified MA portal (dilated to approximately 8‐10 mm wide) and 1 through the AL portal (dilated to approximately 6‐7 mm wide).^6^ The periportal capsulotomy does not generally require capsule closure in femoroacetabular impingement syndrome patients, but in populations at risk for postoperative instability such as patients with joint hypermobility or borderline dysplasia, closure of the periportal capsulotomies may be prudent.^5^, ^13^, ^14^, ^15^ When considering closure of the periportal capsulotomy, the capsulotomy made through the MA portal lies within the iliofemoral ligament and is most important for closure.^13^, ^16^ The capsulotomy made through the AL portal lies in the capsular interval between the iliofemoral and ischiofemoral ligaments and contributes a lesser degree to joint stability compared with the iliofemoral ligament.^8^, ^13^, ^15^, ^16^ However, in cases where there is a high concern for postoperative instability, dual closure of the MA and AL periportal capsulotomies can be performed. We describe our preferred technique for intracapsular dual portal capsule closure of the AL and MA periportal capsulotomies during hip arthroscopy in which closure of the AL periportal capsulotomy is facilitated by passage of the capsule closure suture through the AL portal at the beginning of the case immediately after the capsulotomy is made while the hip is in traction (Video 1).

## SURGICAL TECHNIQUE

**FIGURE 1:**
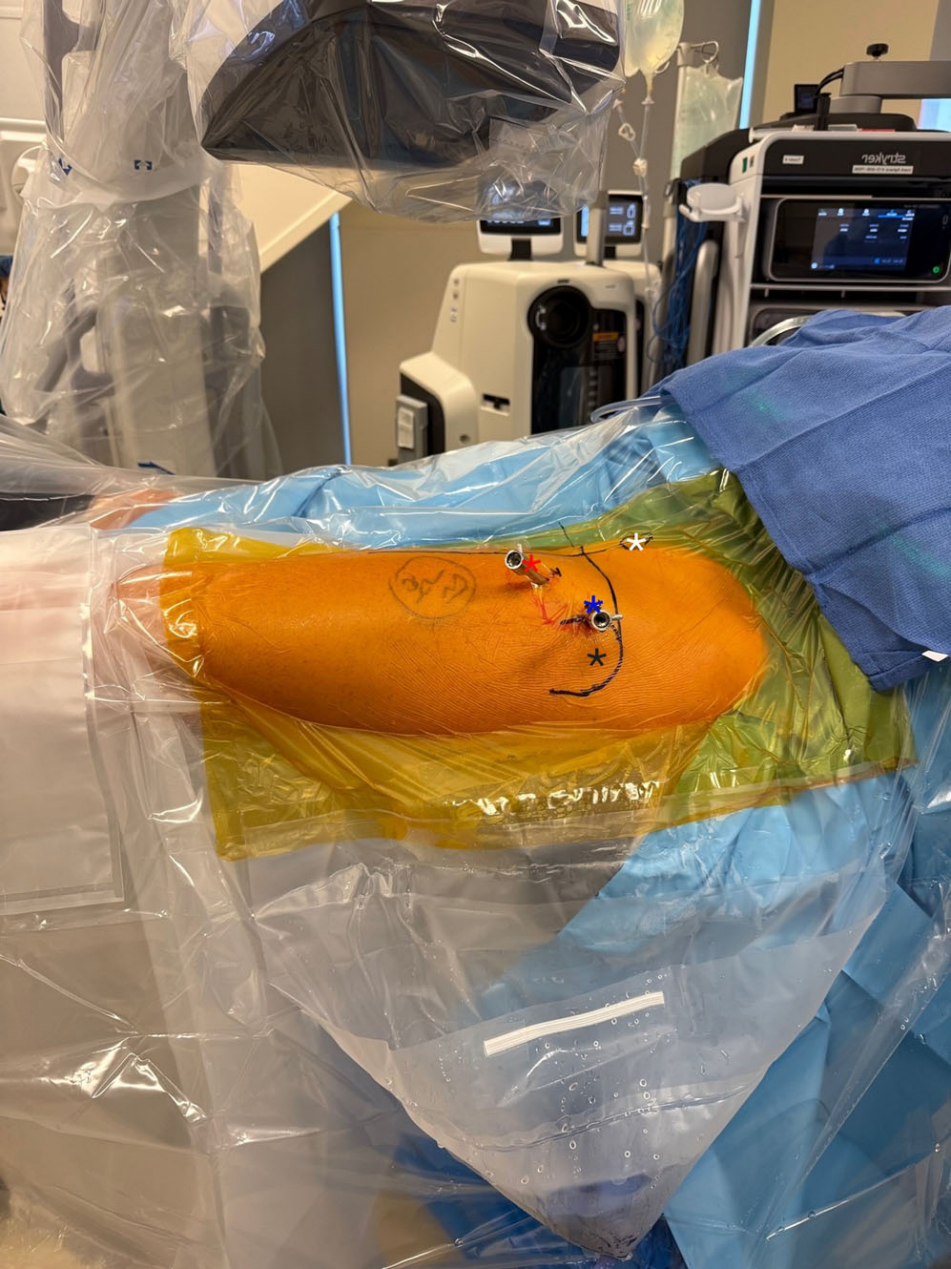
Intraoperative external photo of the left hip positioned supine on operating room table with a postless hip distraction attachment (Guardian Table, Stryker, Kalamazoo, MI) shows our standard setup for hip arthroscopy. The black, white, blue, and red asterisks represent the greater trochanter, the anterior superior iliac spine, the anterolateral portal, and the modified midanterior portal, respectively.

The patient is positioned supine on a standard table with a hip distractor attachment using postless traction (Guardian Table; Stryker, Kalamazoo, MI). To minimize the amount of force needed to distract the hip joint, an air arthrogram is first performed under fluoroscopic guidance.^17^ A standard AL portal is created with fluoroscopy, and a modified MA portal is made using direct visualization, after which 5 mm hip cannulas are placed (Figure 1). Once the portals are created, a radiofrequency ablation device (Arthrocare; Smith & Nephew, Andover, MA) is used to create the periportal capsulotomy by dilating each capsulotomy in line with one another, in the same plane as an interportal capsulotomy. The MA portal is dilated to 8 to 10 mm and the AL portal is dilated to 6 to 7 mm, as previously described.^5^ The diagnostic arthroscopy can then be performed.

**FIGURE 2:**
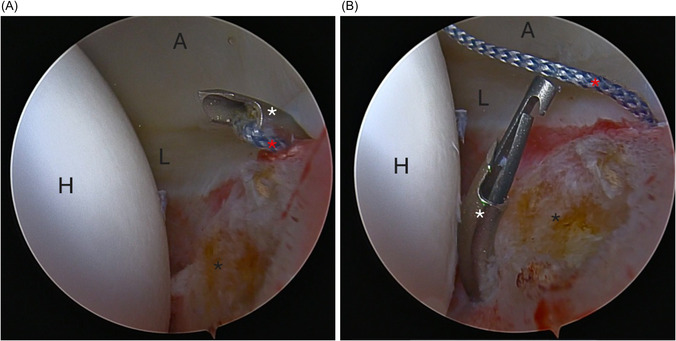
Intraoperative arthroscopic photo during anterolateral portal capsule closure of a left hip positioned supine viewing from the modified midanterior portal with a 70° arthroscope shows (A) passage of a high‐tensile #2 suture across the anterolateral periportal capsulotomy using a 70° Slingshot suture passer (Stryker, Kalamazoo, MI) on the proximal end of the capsulotomy, and (B) the suture retrieved on the distal side of the capsulotomy, creating a simple stitch across the anterolateral portal which will be tied at the conclusion of the surgery. The black, white, and red asterisks represent the anterolateral periportal capsulotomy, suture passer, and #2 suture, respectively. The letters H, L, and A represent the femoral head, labrum, and acetabulum, respectively.

**FIGURE 3:**
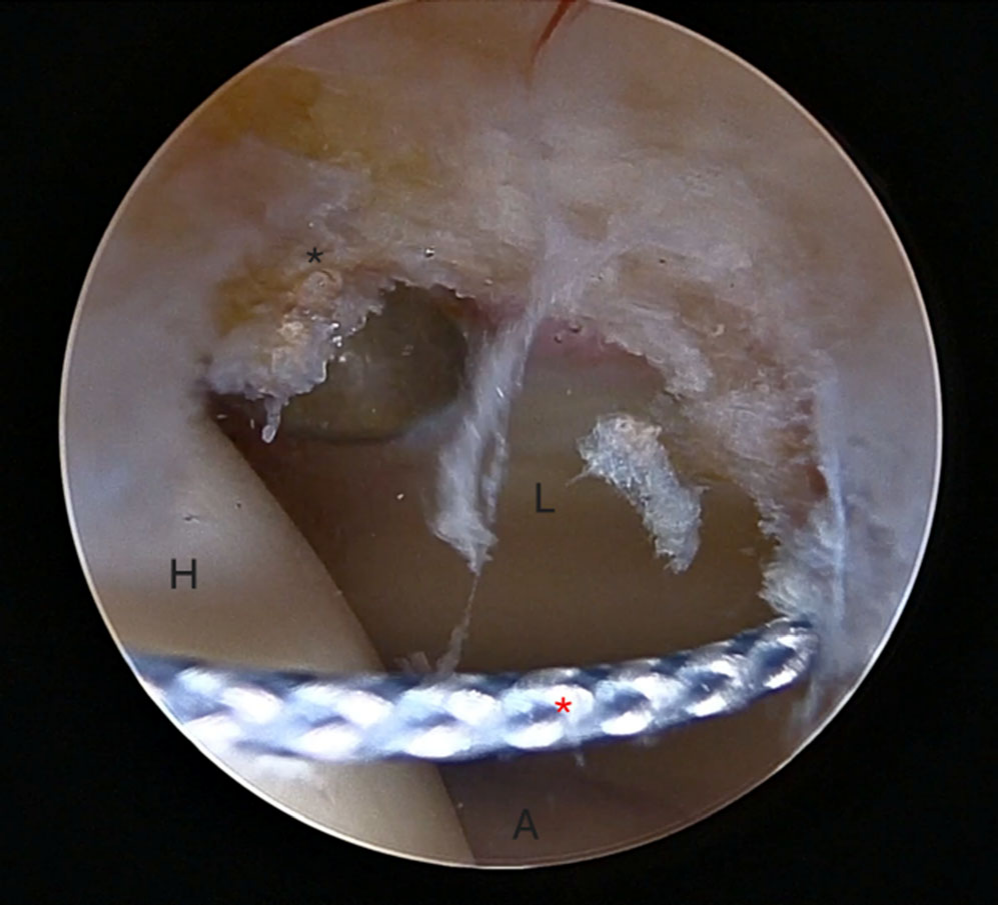
Intraoperative arthroscopic photo during anterolateral portal capsule closure of a left hip positioned supine viewing from the anterolateral portal with a 70° arthroscope shows the high‐tensile #2 suture passed across the anterolateral periportal capsulotomy. The arthroscope is advanced into the joint and the suture will rest beneath the arthroscope for the remainder of the case. The black and red asterisks represent the anterolateral periportal capsulotomy and #2 suture, respectively. The letters H, L, and A represent the femoral head, labrum, and acetabulum, respectively.

**FIGURE 4:**
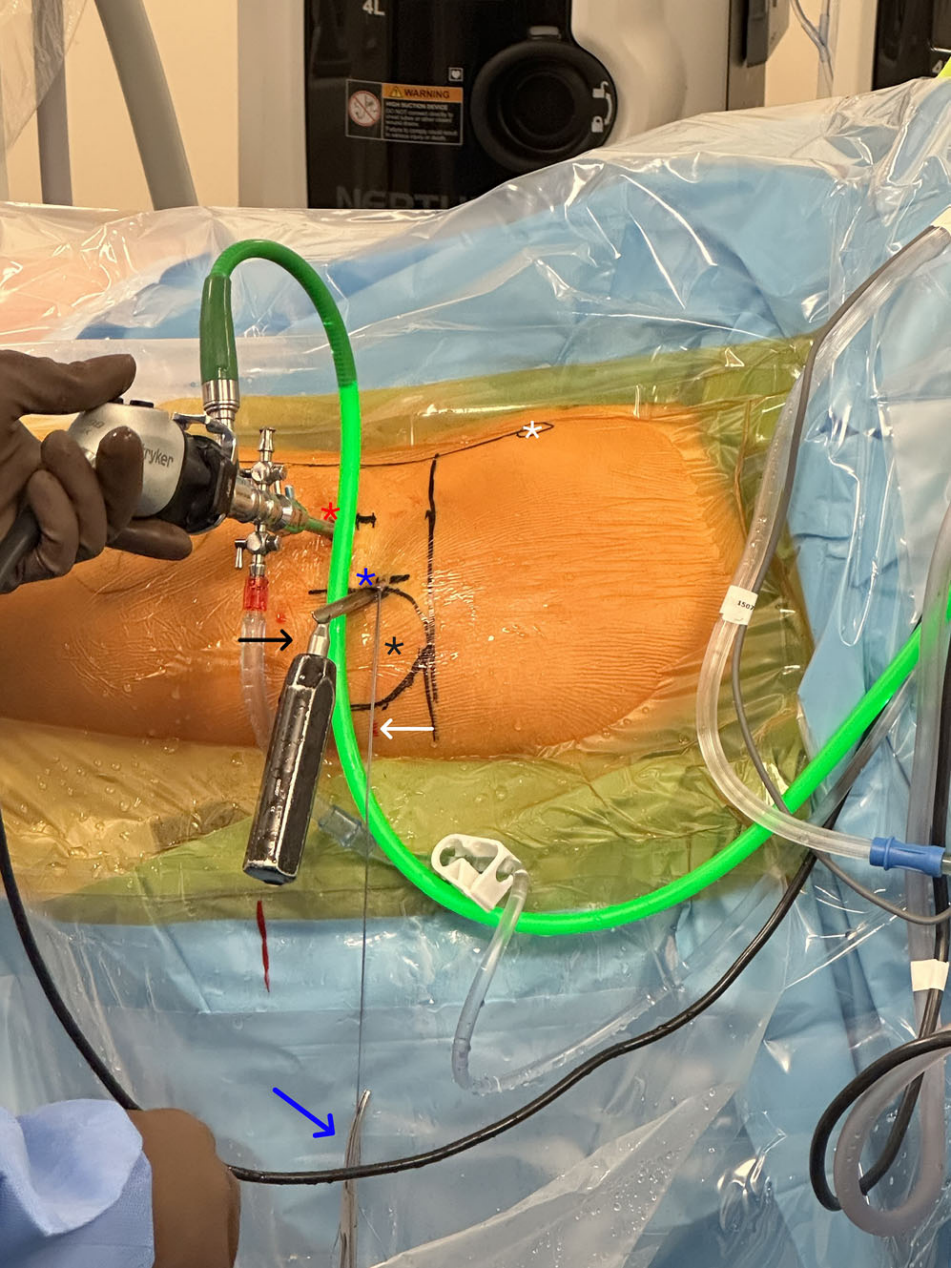
Intraoperative external photo of the left hip positioned supine on operating room table with a postless hip distraction attachment (Guardian Table, Stryker, Kalamazoo, MI) shows the AL portal closure suture clamped outside of the body with the weight of the clamp keeping the suture taught. The black, white, blue, and red asterisks represent the greater trochanter, the anterior superior iliac spine, the anterolateral portal, and the modified midanterior portal, respectively. The black, white, and blue arrows represent the slotted cannula, #2 suture, and clamp, respectively.

After the diagnostic arthroscopy is completed, the suture for repair of the AL periportal capsulotomy is placed prior to any other central compartment procedures. The 70° arthroscope is transferred from the AL to the MA portal, a switching stick is inserted into the AL portal, and the hip cannula is exchanged for a 5.5 mm slotted cannula or “sled.” The sled allows for a 70° suture passer (Slingshot; Stryker, Kalamazoo, MI) loaded with a #2 high‐tensile suture (Orthocord; DePuy Synthes, Warsaw, IN) to be inserted through the AL portal. With the hip in traction, the suture passer is first passed through the proximal end of the capsulotomy adjacent to the labrum, and the suture is released. The suture passer is then passed through the distal end of the capsular tissue and the suture is retrieved (Figure 2A,B). This creates a simple stitch across the capsulotomy (Figure 3). This can be completed under direct visualization from the intracapsular side of the joint through the MA portal. The suture is then clamped outside of the body for later tying with the weight of the clamp keeping the suture taught (Figure 4). The sled is exchanged for the hip cannula again using a switching stick, and the arthroscope is placed back in the AL portal. The suture for the AL portal closure will be tensioned underneath the arthroscope by the weight of the clamp and the remainder of the hip arthroscopy procedure can be completed, including the labral repair, acetabuloplasty, and femoroplasty.

**FIGURE 5:**
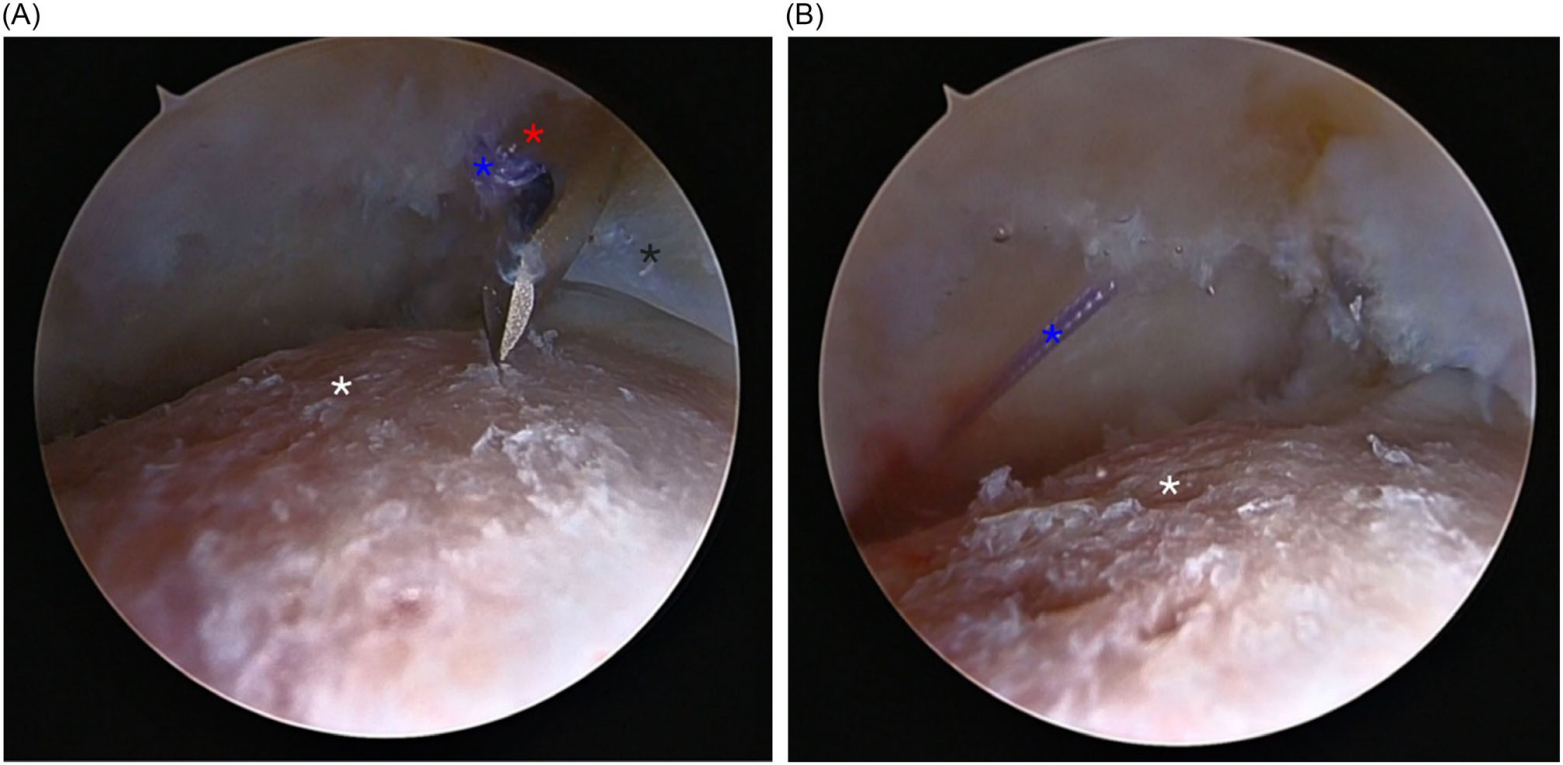
Intraoperative arthroscopic photo of a left hip positioned supine during modified midanterior portal closure after femoroplasty completion viewing from the anterolateral portal with a 70° arthroscope shows (A) penetration of the capsule adjacent to the labrum with a Slingshot suture passer (Stryker, Kalamazoo, MI) for passage of a high‐tensile #2 suture across the modified midanterior periportal capsulotomy and (B) after passage of the suture across the modified midanterior periportal capsulotomy. The stitch is then tied with an arthroscopic knot pusher, closing the periportal capsulotomy while viewing intracapsularly from the AL portal. The red, blue, black, and white asterisks represent the suture passer, #2 suture, the acetabular labrum, and femoroplasty, respectively.

After completion of the hip arthroscopy procedure, the MA portal capsulotomy is closed as previously described.^16^ The 8 × 90 mm disposable cannula (Smith & Nephew, Andover, MA) in the MA portal that was used for labral repair and osteoplasty is retracted until it is superficial to the capsule. The same 70° Slingshot suture passer is placed through the disposable cannula and used to pass a #2 high‐tensile suture through the MA capsulotomy while the arthroscope visualizes intracapsularly from the AL portal. The suture passer is first passed through the proximal end of the capsulotomy, releasing the suture, followed by suture retrieval through the distal end of the capsulotomy as the tissue is more mobile distally (Figure 5A,B). The suture is then retrieved through the disposable cannula and alternating half hitches are tied before the suture is cut with a closed‐ended (tail) cutter.

**FIGURE 6:**
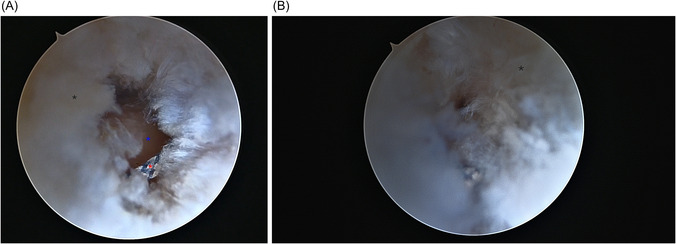
(A) Intraoperative arthroscopic photo of a left hip positioned supine viewing from the anterolateral portal with a 70° arthroscope shows anterolateral portal closure. (B) The arthroscope has been retracted superficial to the capsule, viewing the periportal capsulotomy with the high‐tensile #2 suture pulled taught, showing good approximation of the anterolateral periportal capsulotomy. The blue, black, and red asterisks represent the intra‐articular space, the distal capsular tissue, and the #2 suture, respectively.

**FIGURE 7:**
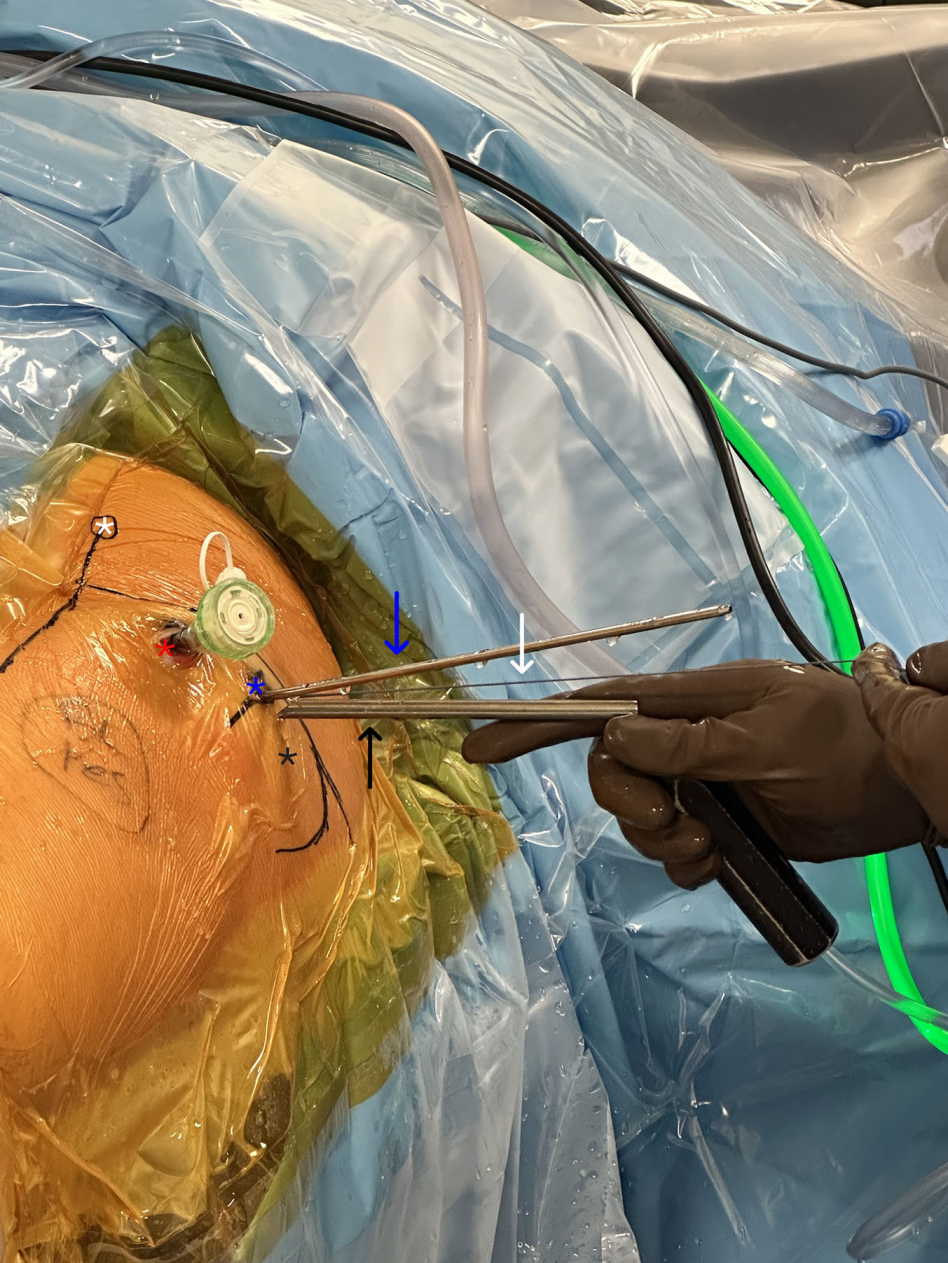
Intraoperative external photo of the left hip positioned supine on operating room table with a postless hip distraction attachment (Guardian Table, Stryker, Kalamazoo, MI). The arthroscope is removed and the hip cannula can be exchanged for a slotted cannula over a switching stick to aid in tying of the AL portal suture. The black, white, blue, and red asterisks represent the greater trochanter, the anterior superior iliac spine, the anterolateral portal, and the modified midanterior portal, respectively. The black, white, and blue arrows represent the slotted cannula, #2 suture, and switching stick, respectively.

Finally, the AL capsulotomy is closed. The suture that was passed at the beginning of the case is now unclamped and pulled taught, confirming good capsular approximation (Figure 6A,B). The arthroscope is then removed from the AL portal, and the 5 mm hip cannula can either be exchanged for a slotted cannula over switching stick to aid in tying or simply removed (Figure 7). Alternating half hitches are tied with a knot pusher, closing the AL capsulotomy in blind fashion. The suture is then cut with a closed‐end “tail” cutter.

### Postoperative Rehabilitation

Capsule closure does not change our postoperative hip arthroscopy protocol. No brace is used. Patients are kept touchdown weight bearing for 1 to 2 weeks, after which they wean off crutches. Low impact exercise and strengthening begins at 6 weeks postoperatively, and running begins at 3 months with return to sports by 6 months.

## DISCUSSION

Various approaches to capsular management and subsequent closure have been described for hip arthroscopy including puncture, periportal, interportal, and T capsulotomy. Performing an appropriately placed capsulotomy provides the visualization and instrument mobility needed to safely perform the procedure, and closure of the capsulotomy facilitates subsequent capsular healing. In our technique, we describe a simple and efficient method for capsular closure of both the AL and MA periportal capsulotomies.

The main advantage of this technique is that it allows closure of both periportal capsulotomies made to access the hip joint to improve capsular healing. Closure of the AL capsulotomy is facilitated by passage of the capsule closure suture through the AL portal at the beginning of the case, immediately after the capsulotomy is made and while the hip is in traction. Passing the AL repair suture while under traction is technically less demanding and minimizes the risk of iatrogenic cartilage or labrum damage compared with passing the suture at the end of the case when traction is no longer applied. Another advantage of this technique is that intracapsular passage of the suture does not require any dissection of the pericapsular bursa superficial to the capsule. This may reduce postoperative pain by minimizing irritation of the pericapsular bursa and limit seroma formation by closing off the tract to the intra‐articular space. This technique adds minimal time to the surgical procedure, may further minimize the risk of postoperative instability, and is relatively inexpensive, with the main cost coming from the use of a disposable 70° suture passing device. Finally, this technique can also be utilized to close the far lateral extension of an interportal capsulotomy (Table 1).

**TABLE 1:** Advantages and Disadvantages of Dual Intracapsular Periportal Capsulotomy Closure

Advantages	Disadvantages
Capsular closure ensures the capsular tissue is reapproximated to facilitate healing after surgery	Passing the AL capsular closure suture while on traction increases total traction time
Performing capsule closure from intracapsular without performing extracapsular dissection may minimize the risk of seroma formation and limit postoperative pain by not irritating the pericapsular bursa	Passing the AL portal suture passer through the capsular tissue directly adjacent to the femoral head and acetabular labrum puts these structures at risk for iatrogenic injury if care is not taken
Passing the AL portal capsule suture while on traction at the beginning of the case is more efficient, less technically demanding and reduces the risk of iatrogenic cartilage and labral damage compared with performing closure at the end of the case while off traction	Placing the AL portal suture at the beginning of the case and tying at the end may increase risk for infection if the suture becomes contaminated during a long procedure
The use of a slotted cannula obviates the need for additional disposable cannulas and minimizes trauma to the overlying muscle	The anterolateral capsular closure suture is at risk for inadvertent transection by the radiofrequency ablation wand, shaver, or burr due to its location near the opening of the AL capsulotomy
Closing both AL and MA portals may reduce the risk of postoperative instability and microinstability in select patient populations	Blind tying of the AL capsule suture may lead to risk of loose knots depending on surgeon skill‐level and comfort with arthroscopic knot typing
This technique can also be utilized to close the far lateral extension of an interportal capsulotomy	

AL, anterolateral; MA, midanterior.

The disadvantages of this technique include the risk of iatrogenic cartilage damage to the femoral head and acetabular labrum, and the slight increase in traction time for passing the AL capsular closure suture. In addition, while passing the AL closure suture at the beginning of the case while under traction may be technically less difficult, it does put the suture at risk for inadvertent damage by the shaver, burr, or radiofrequency device during the remainder of the case. There is also a theoretical increased risk of infection if the suture is contaminated outside of the patient during a long procedure before it is tied off at the end of the case. Pearls and pitfalls for this technique are listed in Table 2.

**TABLE 2:** Pearls and Pitfalls of Dual Intracapsular Periportal Capsulotomy Closure

Pearls	Pitfalls
It is important to continuously monitor time under traction to reduce risk of intraoperative complications	Dilating the anterolateral periportal capsulotomy immediately adjacent to the labrum may not leave sufficient proximal capsule tissue for closure or lead to inadvertent damage to the labrum
When dilating the AL periportal capsulotomy, leave at least 3 mm of proximal capsule adjacent to the labrum to allow for easier suture passage and robust capsular tissue for closure	If multiple passes must be made while attempting to capture the capsular tissue for closure, iatrogenic damage to the capsular tissue, labrum, or femoral head may occur
When passing the capsular closure suture through the AL capsulotomy, first pass suture through the proximal side of the capsulotomy and ensure at least a 2 cm length of suture is passed to allow for easier suture retrieval from the distal end of the capsulotomy	Lack of tension on the anterolateral capsule repair suture during the remainder of the hip arthroscopy procedure can lead to inadvertent damage of the suture with the radiofrequency ablation device, shaver, or burr
Place a free‐hanging clamp on the anterolateral capsule repair suture will put tension on the sutures and help prevent the suture from obstructing the arthroscopy in the AL portal	
While using a slotted cannula to pass the AL capsular repair suture, avoid withdrawing the Slingshot suture passer completely from the wound between passage and retrieval will prevent a tissue bridge	

AL, anterolateral.

In conclusion, this technique for dual portal intracapsular closure of periportal capsulotomy provides a simple and effective method to facilitate capsular healing in populations at risk for postoperative instability such as those with borderline dysplasia or joint hypermobility.

## DISCLOSURES

The author declares the following financial interests/personal relationships which may be considered as potential competing interests: A.L.Z. reports a relationship with Stryker that includes consulting or advisory; reports a relationship with DePuy Synthes Mitek Sports Medicine that includes consulting or advisory; reports a relationship with CONMED Corporation that includes consulting or advisory; and reports a relationship with *Arthroscopy* that includes Associate Editor. The other authors (B.L., J.M.S., N.N.W.) declare that they have no known competing financial interests or personal relationships that could have appeared to influence the work reported in this paper.
